# Residents’ satisfaction and suggestions to improve nephrology residency in Italy, and comparison with the organization in other European countries

**DOI:** 10.1007/s40620-024-01901-2

**Published:** 2024-03-16

**Authors:** Adolfo Marco Perrotta, Silverio Rotondi, Maria Amicone, Irene Cirella, Rossella Siligato, Simone Fontana, Carmen Sivo, Anna Rita Vestri, Giovanni Gambaro, Giorgina Barbara Piccoli, Sandro Mazzaferro

**Affiliations:** 1 Department of Translational and Precision Medicine, Nephrology Unit, Sapienza University of Rome, 00185 Rome, Italy; 2 Department of Public Health, “Federico II” University of Naples, 80131 Naples, Italy; 3 Department of Medicine, Nephrology, Dialysis and Transplantation Unit, University of Padova, 35128 Padova, Italy; 4 Department of Clinical and Experimental Medicine, Unit of Nephrology and Dialysis, University of Messina, 98125 Messina, Italy; 5 Nephrology and Dialysis Unit, San Raffaele Hospital, Vita Salute University, 20158 Milan, Italy; 6 Department of Emergency and Organ Transplantation, Section of Nephrology, University of Bari, 70121 Bari, Italy; 7 Department of Public Health and Infectious Diseases, Sapienza University of Rome, 00161 Rome, Italy; 8 Department of Medicine, University of Verona, 37100 Verona, Italy; 9 Nephrologie, Centre Hospitalier Le Mans, Le Mans, France

**Keywords:** Nephrology, Medical school, Resident, Satisfaction, Questionnaire

## Abstract

**Background:**

In Italy, nephrology residency is available in twenty-one nephrology schools, each with its own strengths and weaknesses. The present study is aimed at exploring the residents’ satisfaction with their training programs.

**Methods:**

Between April 20th and May 19th, 2021, a questionnaire on residency satisfaction consisting of 49 items was sent to 586 residents and 175 recently certified specialists (qualified to practice as nephrologists in 2019 and 2020), with a response rate of 81% and 51%, respectively.

The teaching organization was contextualized with a survey involving 13 European nephrology schools.

**Results:**

Most residency fellowship programs received a good rating with regard to “satisfaction”, in particular for the following items: number of hospitalizations followed-up, chronic hemodialysis training, follow-up of transplanted patients, diagnosis and treatment of glomerulonephritis. The teachings that were identified as being of lower quality or insufficient intensity included vascular access management, ultrasound diagnostics and renal nutrition. The need for improvement in formal teaching programs was underlined. Young nephrologists were rather satisfied with their salary and with the quality of the work they were doing, but only few were interested in an academic career since it was generally held that it is “too difficult” to obtain a university position. Many young nephrologists who filled in the questionnaire felt that lack of experience in peritoneal dialysis and vascular access management was a barrier to finding an ideal job. Compared to other European training programs, the Italian program differs with regard to longer exposure to nephrology (as compared to internal medicine), and greater flexibility for internships in different settings, including abroad.

**Conclusions:**

This first nationwide survey on the satisfaction of residents in nephrology indicates that, despite rather good overall satisfaction, there is room for improvement to make nephrology a more appealing choice and to fulfill the needs of a growing number of renal disease patients.

**Graphical abstract:**

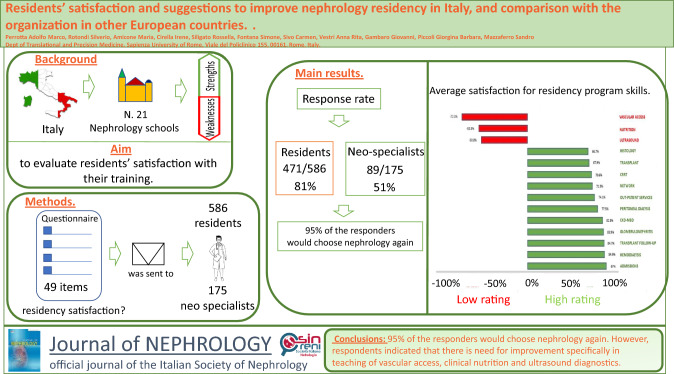

**Supplementary Information:**

The online version contains supplementary material available at 10.1007/s40620-024-01901-2.

## Introduction

“To Be, or Not to Be a Nephrologist” is the title of a recent article by Moura-Neto [1] based on the results of questionnaires filled in by students, residents and professors of medicine in the United States and other countries. In this paper, the author analyzes a number of issues in U.S. medical school programs and seeks to understand why few students are now choosing nephrology. Similar trends have emerged in other countries: in the U.K., for example, medical school graduates’ unwillingness to specialize in nephrology has led to the term *nephrophobia* [2].

Nephrology residency programs vary significantly from country to country. In the United States, the Accreditation Council for Graduate Medical Education (ACGME) sets requirements, including training goals. In addition, after a mandatory period of 3 years in an ACGME accredited internal medicine residency program, the course duration differs, depending on the resident's career path. For example, two years are required to become a clinical nephrologist, but those pursuing a career in research must complete two additional years of training, while qualifying as a transplant nephrologist entails one further year of residency [3].

In Italy, until recently, it was up to each university with a fellowship program in nephrology to decide how to organize it. As a result, different schools had different strengths, but also unavoidable weaknesses. To standardize the requirements for the Italian residency programs, in 2017 the Italian Ministry of University and Research introduced an accreditation process which involves an annual assessment to determine whether the requirements are met (ranging from how many hours a resident is on duty to student–teacher ratios) [4]. Also since 2017, admission to an Italian residency program is based on a nationwide test, in which students choose the different specialties according to their individual ranking. All Italian nephrology residency programs last four years, and fellows sign a contract with the university which specifies salary, working conditions and benefits. As a general rule, fellows rotate among nephrology wards (12 months), out-patient services (12 months, including ultrasound diagnostics) and dialysis (12 months, including peritoneal dialysis). The remaining year is planned locally for activities including kidney transplantation or internal medicine. In addition, upon request, the school’s council can grant permission for internships in other hospitals or research centers in Italy or abroad, for a maximum duration of 3 semesters.

The Ministry recommends that universities create a partnership with nephrology units in their area in order to set up a regional “educational network” integrating practical experience and theoretical knowledge. Despite attempts to standardize these programs, there are significant differences across the current twenty-one Italian nephrology fellowship programs.

The aim of our study was to evaluate residents’ satisfaction with their training.

To achieve this aim, we sent a questionnaire to nephrology residents and to recently certified nephrologists. Participants were asked which changes they would make in the core curriculum and how they felt about their opportunities for employment. The graduates were asked whether and when they had found employment after residency. The answers were reviewed in a broader European context.

## Methods

### National survey

This national survey on the perceived quality of nephrology residency in Italy was designed on behalf of the Italian Society of Nephrology by a group of six nephrology residents from six different schools (see Authorship). The questions were defined by subsequent brainstorming sessions involving the 6 participants, and the final format was agreed upon by all.

The anonymous survey consisted of 49 items that explored the following points: quality of teaching in nephrology courses in medical school; motivation that led graduates to choose a nephrology school; expectations about residency; quality of teaching in the residency program; ways the core curriculum in nephrology could be improved. It was mandatory to answer all of the questions.

Eleven additional questions were directed to the newly certified nephrologists and explored their degree of job satisfaction (if they were employed), how they viewed their occupational profile, the possibility of pursuing an academic career, and their experience abroad. The questionnaire is reported in the Supplementary Material.

The questionnaire was created using Google Forms (Google, Mountain View CA, USA) and was made available from April 20th to May 19th, 2021.

The survey was distributed to the residents of the twenty-one schools of nephrology, by a local reference fellow. It was addressed to 586 residents and 175 nephrologists who graduated in 2019 and 2020 from the above reported nephrology programs.

All the Italian nephrology schools participated in the survey.

The aim of this survey was purely descriptive. We summarized categorical data with counts and percentages. To evaluate the differences in qualitative variables we performed the χ2 test.

### International survey

European nephrologists, mostly academic, from different settings were asked to complete a simple survey on the organization of the nephrology residency in their country. The target choice was made on the basis of personal networks (SM and GBP) or according to ERA indications. Participation was on a voluntary basis.

## Results

### Baseline data

The survey was filled in by 481/586 residents (82% response rate) and by 89/175 recently certified specialists (51% response rate) [Supplemental Table 1]. Of all the respondents, 69% were female and 31% were male. The median age of the respondents was 29.4 ± 3.8 years.

Of the respondent residents, 179 (37%) were in their first year, 125 (26%) in their second, 101 (21%) in their third, and 76 (16.0%) in their fourth year of residency. The number of respondents in the first two years was higher than those in the last two years, since the residency grants made available by the Italian Ministry of Health have almost doubled, acknowledging the increase in demand, mainly in response to the COVID-19 crisis. In fact, the number of available positions in the last four years was: 126 in 2017; 129 in 2018; 162 in 2019 and 246 in 2020.

### Reasons why nephrology was or was not chosen

**Fig. 1:**
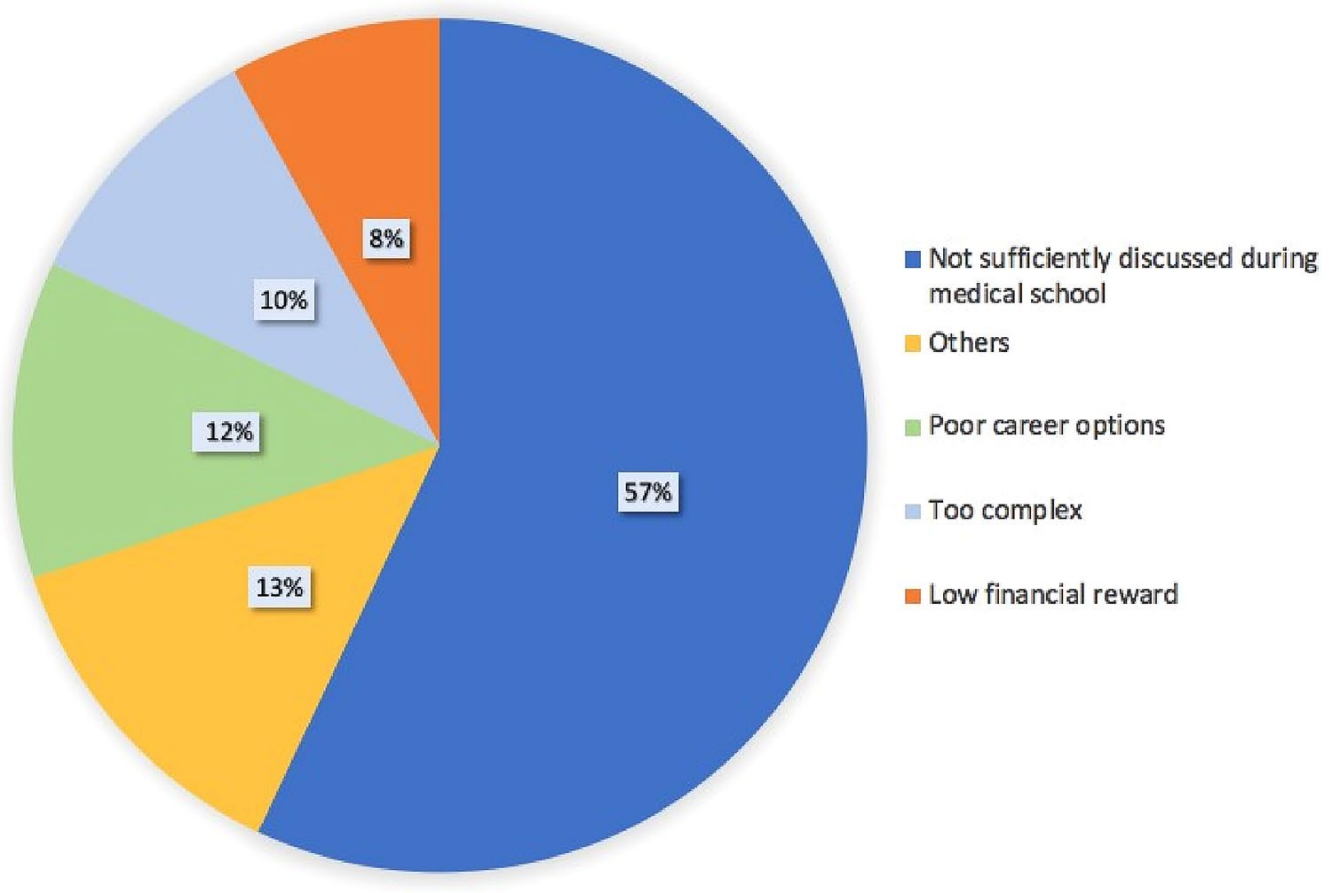
Answers to the question “If nephrology was not your first or second choice, why?”

Only 38% of the participants had sustained a thesis in nephrology. Of the respondents, 51% answered that nephrology was their first choice among the specialties listed in the national test. The reasons why, in their opinion, nephrology was not widely chosen included the low quality of the teaching in medical school (57%), limited career options (12%), and the complexity of the discipline (10%). (Fig. 1).

### Expectations about qualifying as a nephrologist

The majority of participants (80%) answered that the main “hard skills” in nephrology are: nephrological emergencies including hemodialysis (73%), management of electrolyte imbalances (75%), or ultrasound diagnostics (49%).

Asked to identify the “*soft”* skills (defined as the ability to develop fruitful interpersonal relationships with other members of a working team) warranting more consideration, participants chose “problem solving” (79%), “empathy and communicative abilities” (49.8%), and “team work” (38.6%). Asked whether their mentors were interested in teaching *soft skills*, 71% affirmed that they were.

### Quality of the skills acquired in the residency program

Combining “excellent” and “good” scores, the results of the survey were positive for training in the hospital ward (87%), chronic hemodialysis (84.9%), follow-up of transplanted patients (84.7%), and diagnosing and treating glomerulonephritis (83.9%). In particular, higher scores of satisfaction for the skill “follow-up of transplanted patients” were more frequently observed (*p* < 0.021) among the fellows in the last two years compared to those of the first 2 years.

**Fig. 2:**
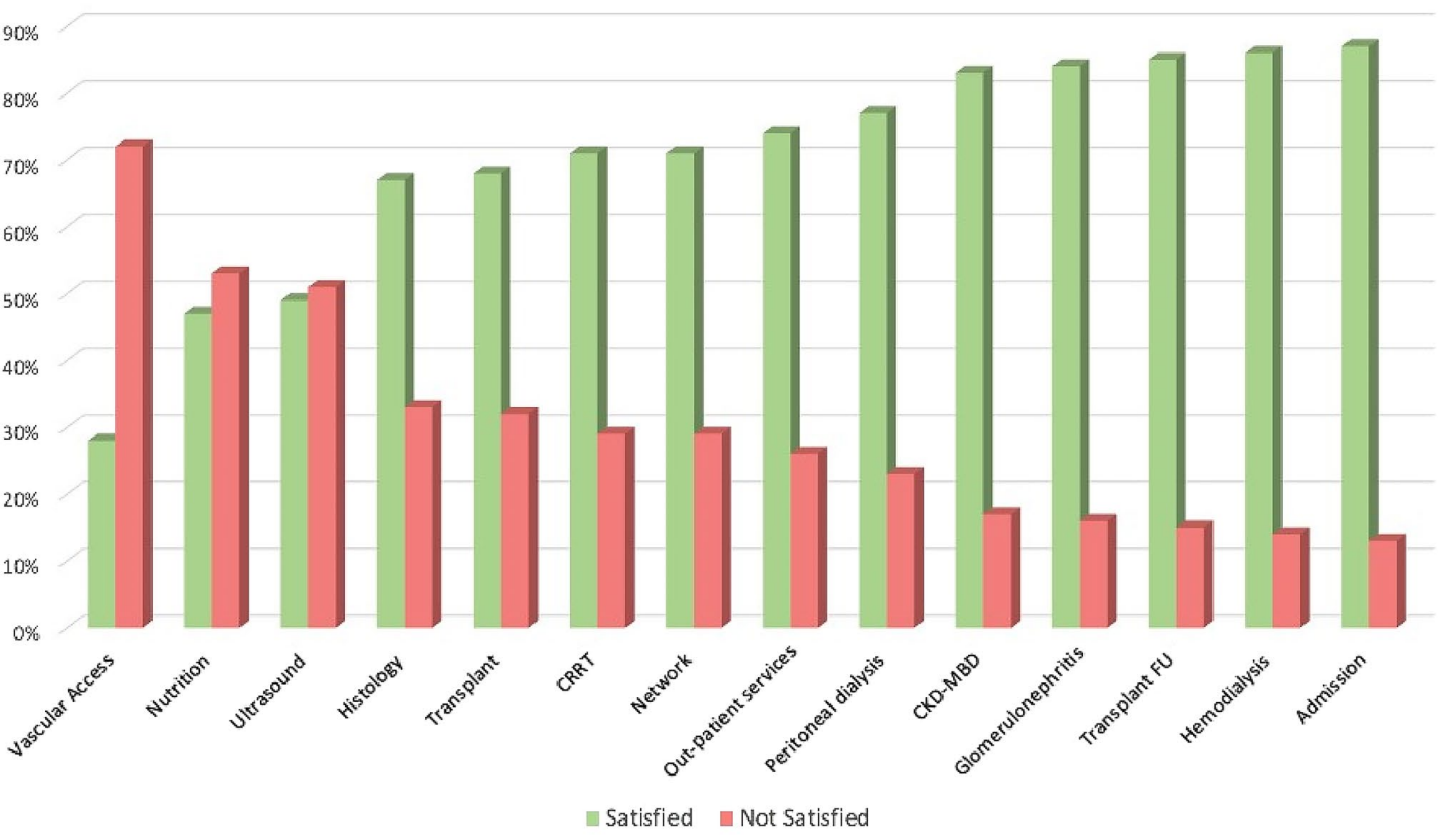
Average satisfaction for residency program skills. *CRRT* Continuous Renal Replacement Therapy, *CKD-MBD* Chronic Kidney Disease—Mineral Bone Disorder

Conversely, “fair” and “poor” satisfaction scores were more frequently reported for management of vascular access (72.1%), ultrasound diagnostics (50.8%) and clinical nutrition (53.5%) (Fig. 2). In particular, vascular access, ultrasound and nutrition received significantly lower scores among the fellows in the last two years (*p* < 0.048; < 0.026 and < 0.004, respectively).

At variance, we found full comparability for all the items of the questionnaire between males and females.

### Teaching quality in the residency program and workload

**Fig. 3:**
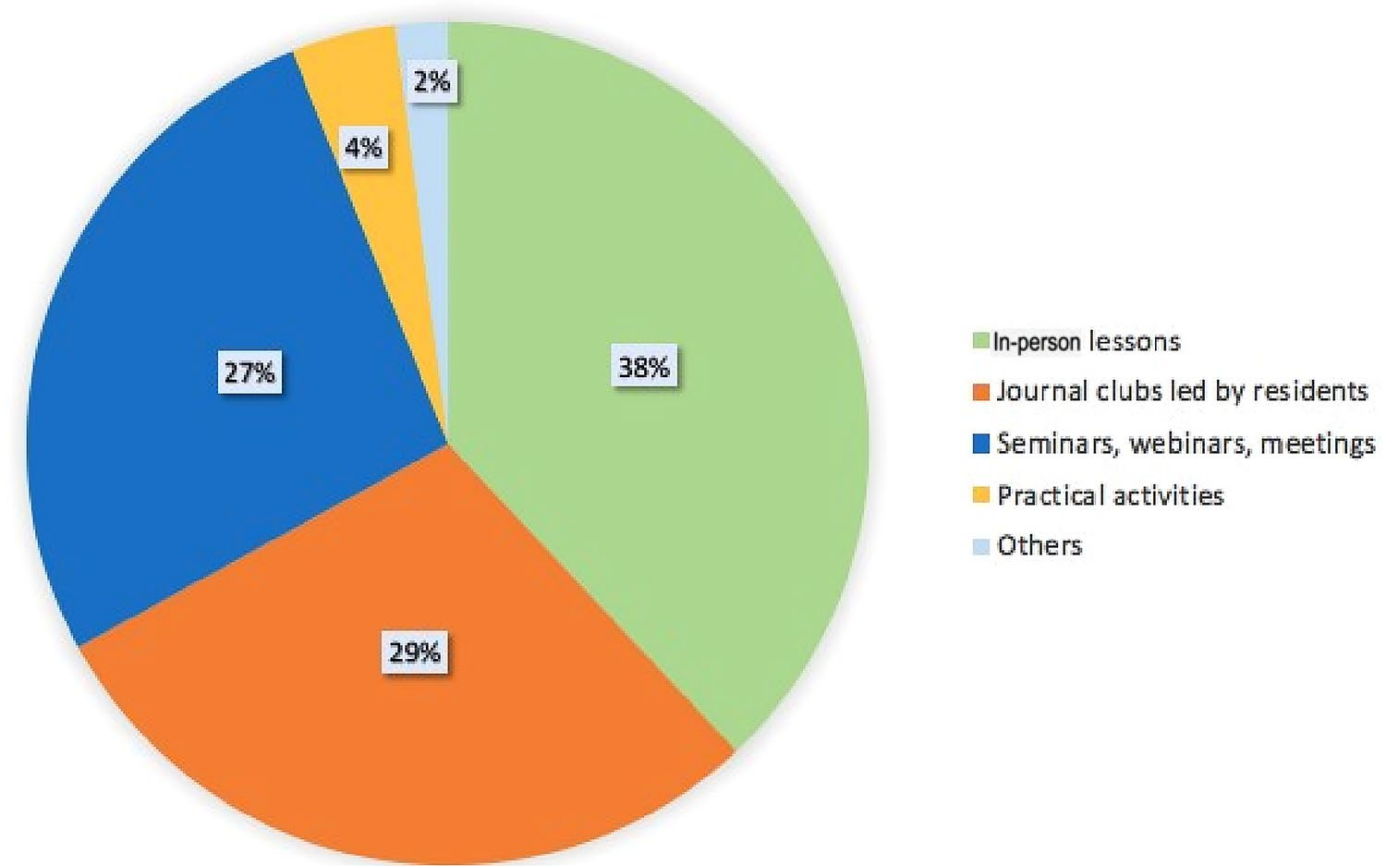
Answers to the question “Which didactic methods do you think are most effective?”

A critical issue was the quality of academic teaching: 37.5% of responders considered lectures essential, but 41% felt that the quality of the lectures they had attended was poor. The preferred educational methods were in-person lessons (38%) seminars (27%), journal clubs or other simulation lab activities. (Fig. 3).

**Fig. 4:**
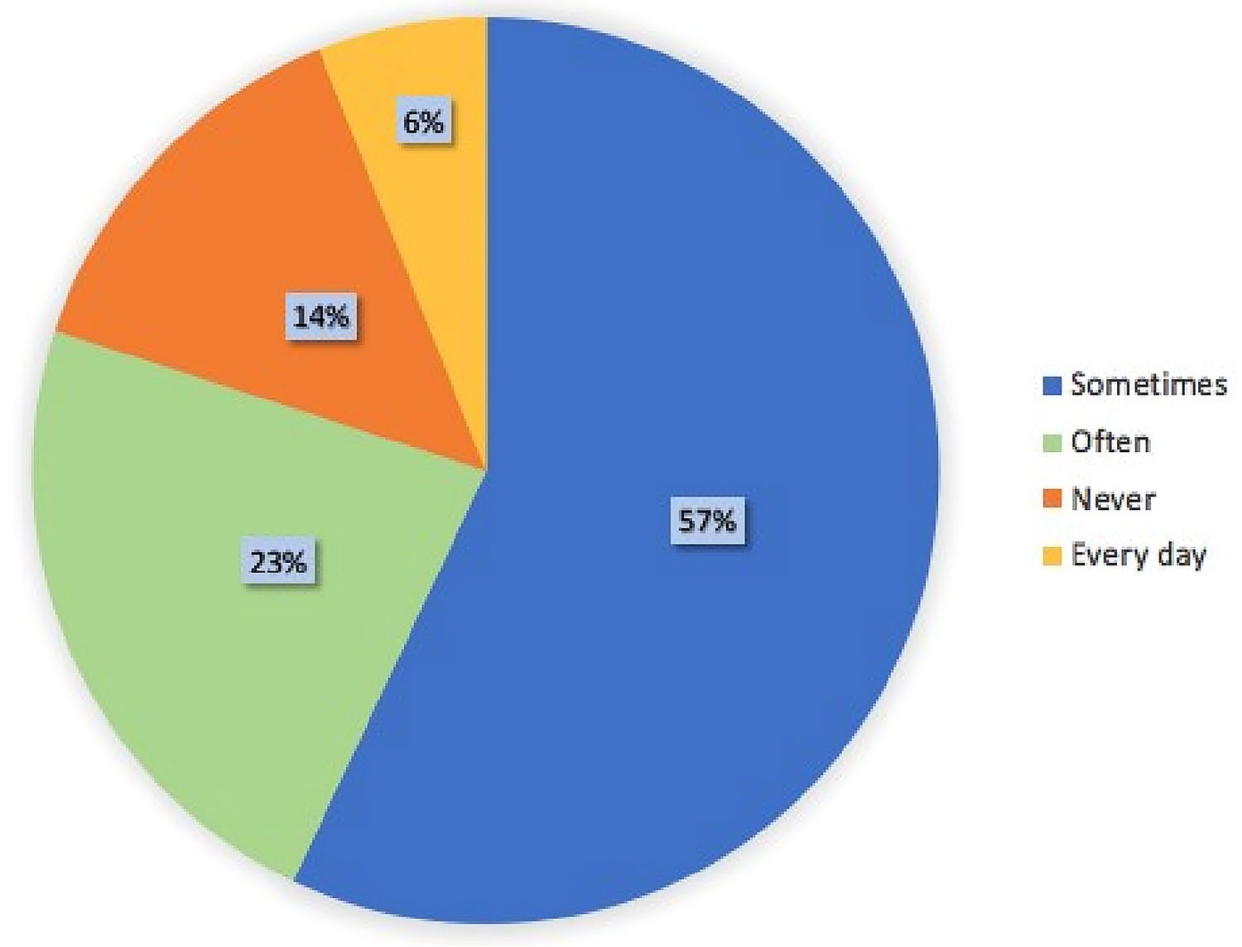
Answers to the question “Have you ever felt tired, depressed or without energy due to the workload?”

Almost two thirds of the respondents indicated that the number of working hours was higher than stipulated in their contract. About one third (29%) of the nephrology residents reported they had experienced some depressive symptoms (Fig. 4).

### Educational needs and suggestions for improvement

Overall, 83% of the residents found that there is room for improvement. Specifically, some topics needed to be better developed, renal nutrition (61%), end of life care (41%), statistics (33.7%), and communication (22.6%).

Although not fully satisfied with their courses, almost all participants (95%) answered that they would choose a nephrology residency again, and 79.6% of them would choose the same university.

### Opinions of the newly certified nephrologists

Of the 175 eligible nephrologists, most of whom were employed in nephrology, 89 (51%) filled in the questionnaire. Almost half (41%) had found a position during their last year of residency, the remaining 49% within one year after graduation, whereas 10% were unemployed. Specifically, 85% were working in the public sector, and 70% were satisfied with their salary. Only 11% were interested in an academic career.

### Baseline determinants of satisfaction with nephrology training.

When we searched for associations between global satisfaction rate (“How do you consider your preparation in relation to the year of the school you are attending?”) and employment rate (“How soon after graduation did you find a job?”) we found no significant association (p = 0.44). Also, there was no relationship between University location (we considered northern, central and southern Italy) and overall satisfaction rate (p = 0.119).

### Differences with other European nephrology training programs.

The organization of the medical schools across European countries differed as for selection modality and criteria, duration and need for a thesis. Selection is based on a national test in Italy, France, Spain and Romania, while direct recruitment is the rule in most other countries. The duration of the training program varies from 4 to 6 years, also based on the previous medical school duration; a final thesis is required in about half of the countries. Spending one or more terms abroad is allowed by only a few countries (Table 1).

**Table 1:** Main characteristics of some residencies in Nephrology in the European wide area

Country	N years	Selection process	Main organization	Evaluation	Thesis	Options for internships in other settings- countries
Italy	4	National test	The students have a”basis” in the university to which the school is affiliated. Some internal medicine internships (cardiology, ICU…) are planned by each school; some time can be spent in non-university centers. Each university center organizes the network of education and care	Oral test at the end of each year	Thesis discussed with the head of the school (university) within a dedicated session	It is allowed a period of three semesters out of the “network”, approved by the board of the school
Austria	5 (2 + 3)	Local application	Twenty-seven months training in “general internal medicine” and then thirty-six months in nephrology	Two national board exams: one at the end of internal Medicine training and one for nephrology	No thesis is needed	Austrian Chamber of Medical Doctors evaluate case-by-case whether training abroad is equivalent to training in Austria
Belgium	5 (3 + 2)	Local application	Nephrology is a specific competency course of 2 years but one of the 2 years can be within the 5 years of internal medicine (thus usually 6 years) Basis in a university (Medical faculty) The rating is based on performance during medical studies, exam at end of studies (30%) and rating during internships	Test after two years in Internal medicine (written), at the end of 5 years in Internal medicine (oral) and at the end of Nephrology school (oral)	No thesis is needed	Highly recommended. Highly variable from faculty to faculty
Croatia	5 (2 + 3)	Local application	Two years internal medicine + three years nephrology. Internal medicine training is set in general hospitals; nephrology at university hospitals	Written test after 2 years for internal medicine; written and oral test at the end of nephrology school	No thesis is needed	Allowed but not mandatory
Czechia	5 (2 + 3)	Local application	Two years of internal medicine with some time in different specialties followed by 3 years of nephrology – partly in regional hospitals under the supervision of a qualified nephrologist and partly in specialized settings (transplantation, glomerular disease)	Written test after internal medicine training and another one at the end of nephrology school	Short thesis (usually case report with the in-depth discussion of one disease) is a part of oral examination	Study abroad is not part of the educational path but it could be allowed on request
Denmark	5	Local application (interview)	Two years at a regional center (focus on internal medicine), two years at a transplant center, one year at the regional center (focus on nephrology). Short theoretical courses on various subjects are planned	No formal tests	No thesis needed. However, for residents without a PhD, a short course and a research paper are part of the program	Optional, but not encouraged. It could be accepted with added time in the program
France	4	National test	The students have a referral university; they have to spend 2–3 semesters in the setting of the school and are free to organize the other semesters in university or non-university centers	No formal tests	Thesis discussed with the head of the school (university) within a dedicated session	One or more semesters allowed out of the specific university network. The option of stages in other countries is not routinely considered
Germany	6 or 5 + 3	Local application	It is mandatory to complete a 5- year general internal medicine residency and additional three years of Nephrology school. Otherwise it is possible to directly apply to a Nephrology division for a combined residency/fellowship program “internal medicine and nephrology”, which lasts six years. It consists of thirty-six months of general internal medicine and nephrology residency (including six months in dialysis and 24 months in the ward), six months each of emergency medicine and intensive care, and 24 months in two different subspecialities of internal medicine	Requirements may vary across regions (Bundesland)	No thesis needed	Work experience in other countries can count for fellowship according to a case-by-case decision of the state authorities (Ärztekammer)
Macedonia, North	6	Local application	The resident provides patient care under the supervision of the attending physician, learning clinical nephrology, and practicing required procedural skills	Oral test of internal medicine at the end of the second year, then oral test of nephrology covering all main aspects	Thesis discussed with the mentor	Educational courses in other countries of maximum three months have to be approved by the coordinator of internal medicine
Netherlands	4 + 2 (internal medicine + nephrology)	Local application	Students apply for a training program in internal medicine (6 years). In the last 2 years of this program, they can choose to specialize in nephrology. During these 2 years, they have 20% internal medicine and 80% nephrology. In the nephrology part, they have mandatory internships (e.g. dialysis, transplantation, consultations, general nephrology) The training program can be in the university hospital, but most of the students will also take six months in a non-university teaching hospital	No formal exam	No thesis needed	May be considered upon request
Romania	5	National test	The students have a”basis” in one of the university hospitals. During the first 2 years, there are some internal medicine internships to follow (cardiology, ICU, internal medicine, rheumatology etc.). The organization is highly dependent upon the resources of each university center, which organizes the network of education and care	Written/oral test at the end of each stage	No thesis required	Three semesters abroad are allowed, approved by the board of the school A National Committee evaluate the equivalency of the stages abroad
Spain	4	National test	Trainees “rotate” through different specialties for 1 year and a half (Internal medicine four months, Cardiology three months, Endocrinology two months, Infectious diseases two months, ICU two months, Rheumatology one months, Radiology one months) + Emergency Room (on duty) The remaining years of Nephrology training is usually equally divided among renal transplantation, dialysis and clinical nephrology	Periodic evaluation by tutors	No thesis required	Compulsory in the few training centers without renal transplantation Volunteers have two periods (one month and two months) for a short national or international fellowship related with specific Nephrology topics
Switzerland	3 + 3	Local application (interview)	Three years of internal medicine fellowship is usually required before starting nephrology. Nephrology school lasts three years	Written and oral tests at the end of training	Not required but highly recommended if the trainee is aiming for an academic career	At least six months to do outside the university training center. A period abroad is usually required for those aiming for an academic career
UK	4 or 3 + 2	National application with regional interviews	Trainees ‘rotate’ through teaching hospitals and general hospitals with renal units (each usually one year). They must spend at least 6 months at a transplant center	After 1 year of specialist training	No thesis needed	It is allowed but needs approval and accreditation assessed

## Discussion

Our survey indicates overall good satisfaction regarding the nephrology residency in Italy, even if improvement is needed in some areas, notably training on ultrasounds, vascular access management and renal nutrition (Fig. 2). Furthermore, the answers provided by newly certified nephrologists suggest that teaching was insufficient for peritoneal dialysis and vascular access management.

However, in spite of the overall good satisfaction rate, and with over 90% of the respondents who stated they would choose Nephrology residency again, a relatively high number of positions remained vacant in the last years, and, according to data made available by medical associations, 21% of the residents either did not accept or abandoned the fellowship within the first year of residency [5]. The finding in our survey that the low degree of satisfaction concerning vascular access and ultrasound diagnostics highlights the need to develop specific educational pathways.

Italy has faced a shortage of nephrologists in recent years [6], which calls for an improvement in the perception of nephrology by medical students. Indeed, the crisis of nephrology is not only an Italian problem. In an effort to find the reasons for the decrease recorded between 2002 and 2009 in the United States, Parker et al. interviewed a large number of medical students [7]. Most participants explained that nephrology was a highly complex specialty and that the poor quality of teaching in medical school was a main reason for not choosing it. In our survey, nephrology teaching in the medical schools was felt by residents to be a critical aspect. In fact, many of our respondents felt that, even though they chose nephrology, nephrology was not properly taught in medical school (57%), while others perceived it as too complex (10%), with few possibilities to find work in the field (12%). Among our respondents, nephrology was the first choice for 51% of the applicants, while in the other cases the choice of nephrology was due to not meeting the score requirements for their first choice.

A cross-sectional study by Daniels et al. [8] involving internal medicine residents in two U.S. universities, before and 3 years after the launch of innovative nephrology fellowship programs, indicated long and burdensome shifts (28.1%), frequent and difficult calls (27.6%), few opportunities to perform procedures (27.3%) and low income as negative factors for the choice of nephrology; the latter item was cited more often in the second survey (9% vs 21% respectively, *p* = 0.04). Other concerns related to nephrology reported by internal medicine residents in the U.S. included the lack of progress in the field, high complexity, lack of role models/mentors, and low prestige [9]. In our survey, roughly 30% of residents declared that they felt tired, depressed or without energy often or every day, due to the high workload.

A survey by the American Society of Nephrology (ASN) involving nephrology residents, showed a heterogeneous degree of satisfaction with traineeship programs, particularly in pediatric nephrology, genetic diseases, renal pathology, renal imaging, interventional procedures, home hemodialysis and kidney disease in pregnancy [10].

Similarly, three Spanish surveys conducted among residents in 2004, 2007 and 2012 highlighted more teaching in transplantation and peritoneal dialysis as the main perceived needs [11, 12].

In response to these data and seeking to encourage nephrology residency, the American Society of Nephrology (ASN) organized two programs—ASN Tutored Research and Education for Kidney Scholars (TREKS) and ASN Students and Residents (STARS)—providing intensive learning opportunities and experience for medical students [13]. Three years later, 40% of the 84 participants self-reported increased interest in nephrology practice and research. In France, satisfaction was strongly associated with the quality of in-person teaching, and on site teaching was overall rated as very insufficient (14). Indeed, in our survey, the best-rated teaching modality was the old-fashioned in-person lesson (38%), while practical activities were selected as the first option by only 4% of respondents.

Overall, our results, in line with the literature, suggest that there is a need to improve the core curriculum in nephrology, starting from medical school where the different aspects of nephrology should be better highlighted with in-person lessons and specific practical activities. During residency, to increase the attractiveness of the course and reduce the number of drop-outs, it appears necessary to improve the teaching on clinical nutrition, vascular access and ultrasound imaging and to include topics like statistics and end of life care. Further, it might be helpful to devise a standardized, national detailed “core curriculum in nephrology” to be adopted by all the schools.

The teaching programs in nephrology vary considerably in Europe. The Italian system stems from more nephrology-specific training with a less coded and shorter stay in internal medicine, possibly because of the longer training period in medical school. The teaching programs are more flexible and influenced by local practices, with an option for a rather long period in other centers or abroad (Table 1).

Among our survey’s weaknesses is the fact that responses to the questionnaire, in particular those of residents in the first two years of their fellowship, may have been based more on the perceived local environment than on direct experience. Furthermore, other emerging issues, such as obstetric or geriatric nephrology, were not included.

In conclusion, this first national Italian survey examining nephology residents’ satisfaction indicates that 95% of the responders would choose nephrology again. However, respondents indicated that there is room for improvement specifically in teaching vascular access, clinical nutrition and ultrasound diagnostics. This needs to be acknowledged to increase interest in nephrology, and to recruit nephrologists so as to fulfill the needs of a growing number of renal disease patients.

### Supplementary Information

Below is the link to the electronic supplementary material.

Supplementary file1 (DOCX 15 KB)

Supplementary file2 (DOCX 28 KB)

## Data Availability

All data are available for any checking or request for those interested.
