# Supplementary material for: Residents’ satisfaction and suggestions to improve nephrology residency in Italy, and comparison with the organization in other European countries
Source: J Nephrol. 2024 Mar 16;37(3):611–23. doi: 10.1007/s40620-024-01901-2 (PMC11150286; doi:10.1007/s40620-024-01901-2)
Supplement: Supplementary file 1 — Supplementary file1 (DOCX 15 KB) [file 40620_2024_1901_MOESM1_ESM.docx]

**SUPPLEMENTARY MATERIAL**

| **School of Nephrology** | **Residents (total)** | **Response rate** | **Recently graduated nephrologists** | **Response**  **Rate** |
| --- | --- | --- | --- | --- |
| Bari | 32 | 87% | 12 | 83% |
| Bologna | 36 | 72% | 12 | 66% |
| Brescia | 28 | 75% | 4 | 0% |
| Catanzaro | 15 | 100% | 7 | 100% |
| Chieti | 19 | 100% | M.D. | M.D. |
| Florence | 29 | 86% | 14 | 78% |
| Foggia | 18 | 100% | 7 | 28% |
| Genoa | 23 | 91% | 10 | 60% |
| Messina | 28 | 67% | 6 | 83% |
| Milan ^1^ | 36 | 66% | 11 | 36% |
| Milan ^2^ | 39 | 100% | 7 | 14% |
| Milan ^3^ | 13 | 69% | 3 | 33% |
| Modena and Reggio Emilia | 16 | 75% | 6 | 16% |
| Naples ^4^ | 31 | 54% | 10 | 0% |
| Naples ^5^ | 44 | 100% | 16 | 71% |
| Padua | 35 | 60% | 7 | 71% |
| Parma | 23 | 87% | 5 | 0% |
| Rome ^6^ | 14 | 100% | 5 | 80% |
| Rome ^7^ | 45 | 80% | 18 | 72% |
| Turin | 40 | 80% | 10 | 30% |
| Verona | 22 | 81% | 5 | 0% |
|  | 586 | 81% | 175 | 51% |

Supplemental Table 1. Description of the total number of specialists and neo-specialists for each school and relative “response rate”.

Abbreviation: M.D., missing data

Apex: [1] Bicocca University; [2] Statale University; [3] San Raffaele Hospital; [4] Federico II University; [5] Luigi Vanvitelli University; [6] Catholic University of the Sacred Heart; [7] Sapienza University.
