# Supplementary material for: Residents’ satisfaction and suggestions to improve nephrology residency in Italy, and comparison with the organization in other European countries
Source: J Nephrol. 2024 Mar 16;37(3):611–23. doi: 10.1007/s40620-024-01901-2 (PMC11150286; doi:10.1007/s40620-024-01901-2)
Supplement: Supplementary file 2 — Supplementary file2 (DOCX 28 KB) [file 40620_2024_1901_MOESM2_ESM.docx]

1. At what university did you study/are you studying?

__________________________

1. What year are you enrolled in?
   1. 1
   2. 2
   3. 3
   4. 4
   5. I completed a specialty track medical residency and was awarded a license to practice as a nephrologist within the last 2 years
2. Sex
   1. Male
   2. Female
3. Was your undergraduate thesis on a topic related to nephrology?
   1. Yes
   2. No
4. Why did you decide to specialize in nephrology? (select only one reason)
   1. My undergraduate thesis was on nephrology.
   2. My undergraduate thesis was not on nephrology but I thought nephrology would be interesting.
   3. It was the best option available at the entrance examination.
5. How did you rank nephrology among your choices?
   1. First
   2. Second
   3. Third
   4. Fourth or lower
6. If nephrology was not your first or second choice, why not? (select only one reason)
   1. I thought it would be too complex.
   2. I thought that my salary as a nephrologist would be low.
   3. I thought it would be difficult to find work.
   4. Nephrology was not sufficiently discussed during medical school so I did not have a clear idea of what working as a nephrologist entails.
   5. Other reason (please specify)
7. How was nephrology taught at the medical school you attended?
   1. Lectures
   2. Lectures + compulsory internship
   3. Lectures + discussions of clinical cases
   4. Lectures + optional internship
8. What determined your choice of nephrology school?
   1. The school’s location.
   2. The school has a good reputation. I thought the academic level would be good.
   3. It was the only option available based on my results on the entrance examination.
9. What do you think are the most important aspects of a nephrology residency program ? (more than one alternative is possible)
   1. Enabling the student to develop skills and acquire knowledge in all branches of nephrology.
   2. Making the student an expert in the sub-specialization they have chosen.
   3. Giving the student the opportunity to follow up patients.
   4. Guaranteeing a period of study abroad.
   5. Guaranteeing the student a minimum of four hours a week of frontal lessons (as stipulated in their contract).
   6. Teaching clinical methodology.
   7. Other (please specify)
10. In your opinion, what are the most important hard skills a nephrology residency program should teach? (two options are possible)
    1. Vascular access surgery
    2. Renal biopsy
    3. US diagnostics
    4. Immunosuppressive therapy management
    5. Management of dialysis in chronic and acute patients
    6. Second-level urinalysis
    7. Management of acute patients and infusion therapy
    8. Clinical research
    9. Other (please specify)
11. In your opinion, what are the most important soft skills a nephrology residency program should teach? (two options are possible):
    1. Problem solving
    2. Public speaking
    3. Team work
    4. Liaison skills
    5. Professional integrity
    6. Communicating with colleagues
    7. Empathy and communicating with patients and their families
12. In your school, are/were mentors interested in teaching soft skills?
    1. Yes
    2. No

**EVALUATE YOUR SCHOOL**

**How satisfied are you with your school? Rate each aspect of its training program as excellent, good, fair or poor.**

1. Number of nephrology patients hospitalized per year and variety of case histories:
   1. Excellent
   2. Good
   3. Fair
   4. Poor
2. Number of vascular access surgeries performed as leading operator:
   1. Excellent
   2. Good
   3. Fair
   4. Poor
3. Internship in a transplant center and learning what is entailed in the early phases of renal transplant follow-up (induction therapy and surgery):
   1. Excellent
   2. Good
   3. Fair
   4. Poor
4. Long-term renal transplant follow-up:
   1. Excellent
   2. Good
   3. Fair
   4. Poor
5. US diagnostics:
   1. Excellent
   2. Good
   3. Fair
   4. Poor
6. Specialist out-patient services (ADPKD, urolithiasis, pre-emptive transplants, etc.):
   1. Excellent
   2. Good
   3. Fair
   4. Poor
7. Chronic hemodialysis:
   1. Excellent
   2. Good
   3. Fair
   4. Poor
8. Acute hemodialysis:
   1. Excellent
   2. Good
   3. Fair

21.4. Poor

1. Peritoneal dialysis:
   1. Excellent
   2. Good
   3. Fair
   4. Poor
2. Clinical nutrition:
   1. Excellent
   2. Good
   3. Fair
   4. Poor
3. Management of glomerulonephritis:
   1. Excellent
   2. Good
   3. Fair
   4. Poor
4. CKD-MBD management:
   1. Excellent
   2. Good
   3. Fair
   4. Poor
5. Histopathology:
   1. Excellent
   2. Good
   3. Fair
   4. Poor
6. Involvement in clinical trials:
   1. Excellent
   2. Good
   3. Fair
   4. Poor
7. Cross-disciplinary interaction:
   1. Excellent
   2. Good
   3. Fair
   4. Poor
8. Opportunities to attend local, national and international meetings:
   1. Excellent
   2. Good
   3. Fair
   4. Poor
9. Discussions of recently published scientific articles(journal clubs, etc.):
   1. Excellent
   2. Good
   3. Fair
   4. Poor
10. Educational network:
    1. Excellent
    2. Good
    3. Fair
    4. Poor
11. Did you work in a hospital or other structure that was part of your school’s educational network?
    1. Yes.
    2. No, I wasn’t interested.
    3. No, the school I attended has not an educational network.
12. Frontal lessons:
    1. Excellent
    2. Good
    3. Fair
    4. Poor
13. What teaching methods are used? (maximum 3 options)
    1. Journal clubs led by residents
    2. Seminars, webinars, meetings
    3. Frontal lessons
    4. Skill labs
    5. None of above
14. How often?
    1. Weekly
    2. Monthly
    3. Several times a year
    4. Never
15. Which methods do you think are most effective?
    1. Journal clubs led by residents
    2. Seminars, webinars, meetings
    3. Frontal lessons
    4. Skill labs
16. How much of your working time is spent in educational activities?
    1. <10%
    2. 10%
    3. 20%
    4. >20%
17. How many hours a week do you spend caring for patients?
    1. <32
    2. 32-40
    3. >40
18. Apart from compiling patients’ clinical charts, how much of your working time is devoted to recordkeeping and paperwork (e.g. sending faxes, contacting administrators, etc.)?
    1. 10-20%
    2. 20-30%
    3. 30-40%
    4. 40-50%
    5. >50%
19. Have you ever felt tired, depressed or unenergetic due to the work load?
    1. Never
    2. Sometimes
    3. Often
    4. Every day
20. Based on the what you have been told by residents at other nephrology schools, do you think your school’s training program is comparable to the programs in other schools?
    1. Yes.

41.2. No, I think it is better than at most other schools.

41.3. No, I think it is not as good as at most other schools.

1. How did you obtain information about other nephrology schools?
   1. Participating in conferences.
   2. Talking to people I know who are specializing in nephrology at other schools.
   3. Hearsay.

**PROPOSALS**

1. Do you think the core curriculum should be broader?
   1. Yes
   2. No
2. If you answered yes, which of the following subjects would you add? (more than one is possible)
   1. Nutrition
   2. Statistics
   3. Communication skills
   4. End of life ethics
   5. Bioinformatics
   6. Telemedicine
   7. Other (please specify)
3. Is there is anything else you wish to comment on? Use the space below.

__________________

**CONCLUSIONS**

1. Are you happy that you decided to specialize in nephrology?
   1. Yes
   2. No
2. Are you happy with your choice of school?
   1. Yes
   2. No
3. How do you consider your preparation in relation to the year of school you are enrolled in?
   1. Poor
   2. Fair
   3. Good
   4. Excellent
4. Have you taken part in meetings organized by Italian Society of Nephrology?
   1. Yes
   2. No

**PLEASE ANSWER THE FOLLOWING QUESTIONS IF YOU HAVE COMPLETED YOUR STUDIES AND RECEIVED CERTIFICATION TO PRACTICE AS A NEPHROLOGIST**

1. How soon after graduating did you find a job?
   1. I was offered a position before graduating.
   2. I found work in less than a year.
   3. I have not found work yet.
2. Do you work as nephrologist?
   1. Yes
   2. No
3. Which field of nephrology do you work in?
   1. Hemodialysis
   2. Peritoneal dialysis
   3. Transplants
   4. Out-patient care
   5. Vascular access surgery
   6. Other (please specify)
4. Do you work mainly in the public or private sector?
   1. Private
   2. Public
5. Have you ever been awarded a research grant?
   1. Yes
   2. No
6. After completing your studies in a nephrology residency program, did you want to pursue an academic career?
   1. Yes
   2. No
7. If not, why not?
   1. I’m not interested.
   2. I was unable to find a university position.
   3. I consider salaries inadequate.
   4. Other (please specify)
8. If you answered yes, when did you become interested in a academic career?
   1. I was interested before beginning my specialist training.
   2. I developed an interest in doing research while specializing.
   3. I saw it as an employment opportunity.
   4. Other (please specify)
9. Did you enroll in a PhD program after finishing specialist training?
   1. Yes
   2. No, I wasn’t interested
   3. No, I applied to PhD programs but wasn’t accepted.
10. Are you working abroad or have you worked abroad since finishing your studies?
    1. I studied abroad while completing the nephrology internship.
    2. I worked abroad after graduating.
    3. I am currently working abroad.
    4. I have never worked abroad.
11. Do you consider your salary adequate?
    1. Yes
    2. No
